# Identification of a Novel Immune Landscape Signature for Predicting Prognosis and Response of Colon Cancer to Immunotherapy

**DOI:** 10.3389/fimmu.2022.802665

**Published:** 2022-04-28

**Authors:** Zheng Wang, Jingru Song, Nisma Lena Bahaji Azami, Mingyu Sun

**Affiliations:** ^1^Shanghai University of Traditional Chinese Medicine, Shanghai, China; ^2^Key Laboratory of Liver and Kidney Diseases, Institute of Liver Diseases, Shuguang Hospital Affiliated to Shanghai University of Traditional Chinese Medicine, Shanghai, China

**Keywords:** colon cancer, immune-related gene prognostic index, weighted gene co-expression network analysis (WGCNA), prognosis, signature

## Abstract

**Purpose:**

To construct an immune-related gene prognostic index (IRGPI) for colon cancer and elucidate the molecular and immune characteristics as well as the benefit of immune checkpoint inhibitor (ICI) therapy in IRGPI-defined groups of colon cancer.

**Experimental Design:**

Transcriptional and clinical data of colon cancer samples were obtained from The Cancer Genome Atlas (TCGA) (n = 521). Immune-related genes were obtained from ImmPort and InnateDB databases. 21 immune-related hub genes were identified byweighted gene co-expression network analysis (WGCNA). the Cox regression method was used to construct IRGPI and validated with Gene Expression Omnibus (GEO) dataset (n = 584). Finally, the molecular and immune profiles in the groups defined by IRGPI and the benefit of ICI treatment were analyzed.

**Results:**

8 genes were identified to construct IRGPI. IRGPI-low group had a better overall survival (OS) than IRGPI-high group. And this was well validated in the GEO cohort. Overall results showed that those with low IRGPI scores were enriched in antitumor metabolism, and collated with high infiltration of resting memory CD4 T cells and less aggressive phenotypes, benefiting more from ICI treatment. Conversely, high IRGPI scores were associated with cell adhesion molecules (CAMs) and chemokine signaling pathways, high infiltration of macrophage M1, suppressed immunity, more aggressive colon cancer phenotypes, as well as reduced therapeutic benefit from ICI treatment.

**Conclusions:**

IRGPI is a promising biomarker to differentiate the prognostic and molecular profile of colon cancer, as well as the therapeutic benefits of ICI treatment.

## Introduction

Colon cancer, which is one of the major causes of death worldwide, has become the third most common cancer in men and the second most common cancer in women globally. Approximately 25% of colon cancer patients have been diagnosed with stage IV cancer. Another 25% of the patients with colon cancer are diagnosed in early stages, but their cancer still metasticizes (1). The five-year survival rate for patients with stage IV tumors is less than 10% (2). It is estimated that by 2030, the incidence of colorectal cancer (CRC) will increase by 60%, with more than 2.2 million new cases worldwide, resulting in more than 1.1 million deaths (1). Over the past decades, immunotherapy drugs have been extensively used in the treatment of cancer and shown clinical efficacy. Immune checkpoint inhibitors (ICIs) targeting programmed cell death-1 (PD-1) or cytotoxic T lymphocyte-associated protein-4 (CTLA-4) have achieved tumor regression in several cancers, such as melanoma, lung cancers, and Hodgkin lymphoma (3, 4). Furthermore, PD-1 and programmed cell death-ligand 1 (PD-L1) blockers appear to be a promising option for patients with colon cancer (5). A major limitation, however, is the low response rate of patients with colon cancer to ICI therapy. The tumor immune microenvironment (TME) has gained increasing attention as an indispensable component of immunotherapy (6). In fact, TME may be used as a major prognostic indicator, which in turn can improve precision targeted therapy (7). Hence, Identifying potential prognostic markers associated with the therapeutic benefit of immunotherapy could allow patients with colon cancer to receive more individualized therapies.

In this study, we sought to develop an immune-related gene prognostic index (IRGPI) that is capable of predicting not only the prognosis of conventional therapy but also immunotherapy. We then assessed the molecular and immunological characteristics of IRGPI. Its reliability was validated with multiple datasets, and IRGPI was a promising prognostic biomarker for patients receiving conventional treatment and immunotherapy.

## Material and Methods

**Figure 1:**
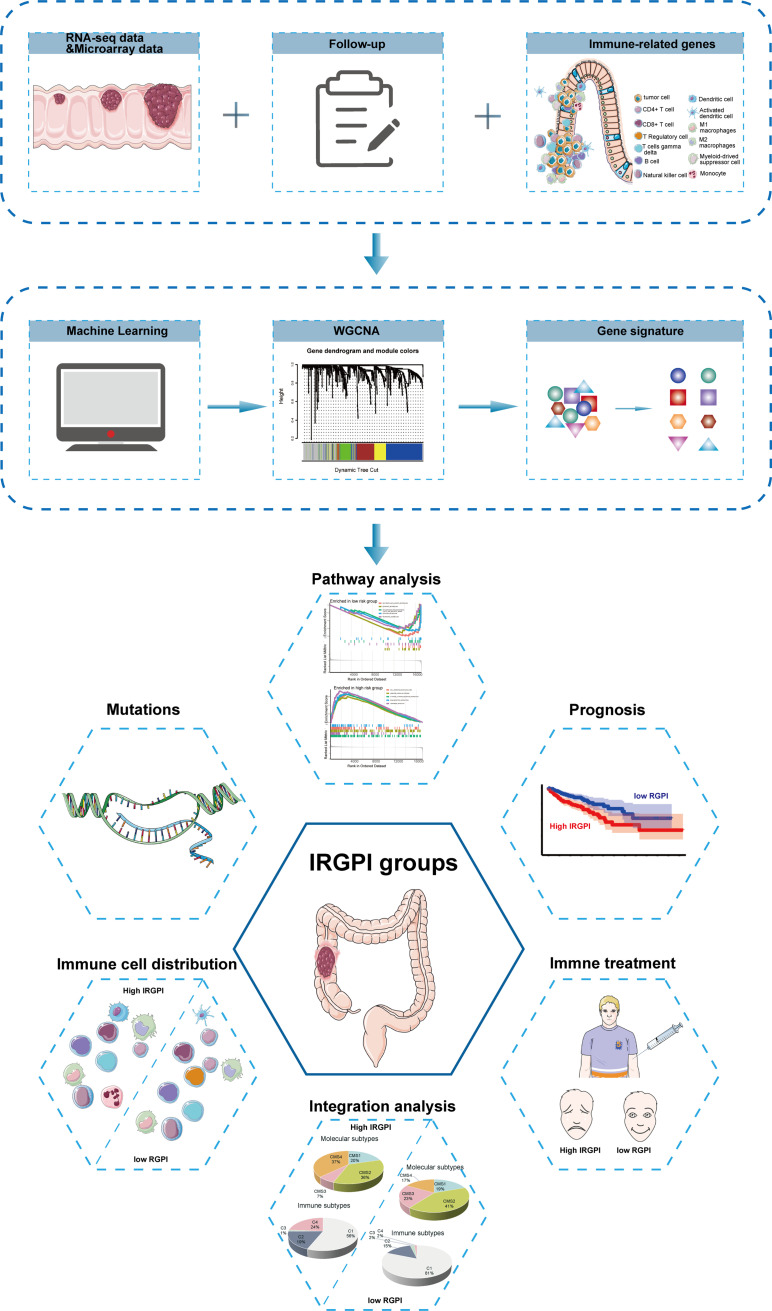
Flow chart of the whole study.

The flow chart of the whole study is presented in **Figure 1**.

### Collection of Sample Information

RNA-seq data of 521 colon cancer samples, including 480 cancer samples and 41 paracancerous tissue samples, and their clinicopathological information were downloaded from the TCGA database (https://portal.gdc.cancer.gov/projects/TCGA). RNA-seq data of 584 colon cancer samples (GSE39582) and the survival information were downloaded from the GEO database (https://www.ncbi.nlm.nih.gov/geo/) (8). The entire TCGA cohort and GSE cohort were used as the testing set and validation set, respectively. The lists of immune-related genes were downloaded from the ImmPort (https://www.immport.org/shared/home) and InnateDB (https://www.innateDBdb.com/) databases.

### Differentially Expressed Immune-Related Genes (DEIRGs)

By analyzing RNA-seq data from colon cancer samples obtained through TCGA, a list of differentially expressed genes (DEGs) (false discovery rate (FDR) < 0.05, |log_2_FC| > 1) for tumor and normal tissues was determined using the limma package (9). The DGEs were compared and crossed with immune-related genes (IRGs) to obtain DEIRGs. The heatmap of DEIRGs was generated using pheatmap package in R. After extracting the list of immune-related genes from ImmPort and InnateDB, DEIRGs were selected and analyzed by Gene Ontology (GO) and Kyoto Encyclopedia of Genes and Genomes (KEGG) analysis using clusterProfiler package of R (10).

We then constructed a co-expression network using the WGCNA package (11) to identify hub genes, as WGCNA analysis is biologically more significant compared with traditional methods (12). All crossover genes were reflected in the co-expression network. Scale-independent and average connectivity analyses were performed on modules with different power values to calculate the software threshold parameters. In this study, we set the scale irrelevance value to 0.9 to identify the hub genes. The adjacency matrix was transformed into a topological overlap matrix (TOM) describing the similarity of gene expression. 1-TOM represented the heterogeneity between genes. Based on the TOM-based similarity, a dynamic tree-cutting algorithm was used to group genes into different modules (clusters). Here, we set the cut height to 0.3 and the minimum module size to 30. The best cut-off value for overall survival (OS) was calculated for each hub gene using survminer and survival R packages (13). 21 immune-related hub genes significantly associated with survival were screened for further analysis (*P* < 0.05). To reveal relevant genetic alterations, somatic mutations of the 21 immune-related hub genes were analyzedusing maftools package in R (14).

### Construction and Validation of the IRGPI

Among the 21 immune-related pivotal genes, those with significant effects on OS were identified. IRGPI was then constructed by multivariate Cox regression analysis. The risk score was calculated as below:


$$Risk\;Score=\sum_{i=1}^{j}coeffient_{i}\times expression_{i}$$

IRGPI was calculated by multiplying the expression data of certain genes for each sample by their weights in the Cox model and then adding them together. The prognostic ability of IRGPI was assessed by Kaplan-Meier analysis in the TCGA and GEO cohorts, respectively. To further verify the prognostic value of IRGPI, univariate and multivariate Cox regression analyses were performed.

### Molecular and Immunological Characterization of Different IRGPI Groups and Comprehensive Analysis of ICI Treatment

First, differential expression analysis of all genes was performed by limma package in R, and samples with high (n = 226) and low (n = 227) IRGPI scores were obtained. Enrichment analysis was then performed by clusterProfiler package in R using KEGG and genomic-based gene set enrichment analysis (GSEA) methods to identify the signaling pathways(*P* < 0.05). Finally, gene mutations in the two IRGPI groups were analyzed using maftools package.

To clarify the immune characteristics of the 480 colon cancer samples, expression data was imported into CIBERSORT (http://cibersort.stanford.edu/) and iterated 1000 times to estimate the relative proportions of 22 immune cell species. We then compared the relative proportions of the 22 immune cell species and clinicopathological factors between the two IRGPI groups, and the results were presented as landscape plots. Moreover, we performed GSEA on certain gene signatures and compared the score between two IRGPI groups in order to understand the immune and molecular functions (15–18).

Widely used biomarkers and other published immune-related signatures for cancer immunotherapy were evaluated to compare them with our IRGPI, in order to determine the prognostic value of IRGPI for patients with colon cancer. We compared the prognostic value among IRGPI, TIDE, and T cell-inflamed signature (TIS) using timeROC package (19) in R. The TIDE score, which can be calculated online (http://tide.dfci.harvard.edu/), predicted the prognosis of patients with tumors treated with first-line anti-PD1 or anti-CTLA4 therapy more accurately than other biomarkers such as PD-L1 levels and mutational load (20) The TIS score, which can be calculated as the mean of the log_2_-scale normalized expression of 18 signature genes (21), can enrich the clinical benefit of immune checkpoint blockade (22). We also selected immune prognostic models constructed in previous studies such as “Zhang signature” (23) and “Deng signature” (24). In addition, ROC curve analysis was performed to obtain the area under the curve (AUC).

### Statistical Analysis

An independent *t*-test was performed for continuous variables between the two groups. Categorical data were tested using the chi-square test. TIDE score between groups was compared by the Wilcoxon test. Univariate survival analysis was performed by Kaplan-Meier survival analysis with the log-rank test. Multivariate survival analysis was performed using the Cox regression model. A two-sided *P* < 0.05 was considered significant.

## Results

### Immune-Related Hub Genes

**Figure 2:**
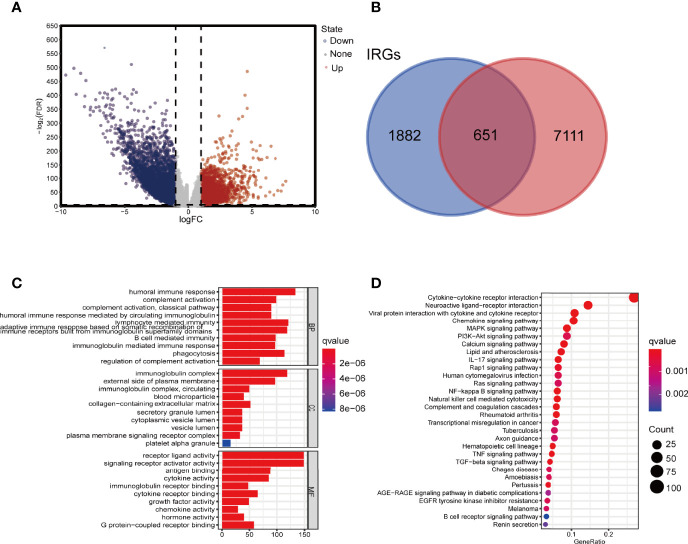
Screening for DEGs and enrichment analysis. **(A)** Volcano map of DEIRGs. **(B)** Venn diagram showing the intersection of DEGs and IRGs. **(C)** GO enrichment analysis of DEIRGs for BP, CC, and MF, respectively. **(D)** KEGG enrichment analysis of DEIRGs. DEGs, differentially expressed genes; DEIRGs, differentially expressed immune-related genes; IRGs, immune-related genes; GO, Gene Ontology; BP, biological process; CC, cellular component; MF, molecular function; KEGG, Kyoto Encyclopedia of Genes and Genomes.

In differential expression analysis (480 tumors vs. 41 normal tissue samples), a total of 7762 DEGs were screened (**Figure 2A**). By crossing these genes with the list of immune-related genes, 651 differentially expressed immune-related genes were obtained. Compared with genes in normal tissue samples, 257 genes were upregulated and 394 downregulated in the tumor samples (**Figure 2B**).

The top 10 GO terms and 20 KEGG pathways were shown in **Figures 2C, D**. Functional enrichment analysis revealed that the most relevant signaling pathway for DEIRGs was “cytokine-cytokine receptor interaction”. The most enriched term in biological process (BP), molecular function (MF), and cellular component (CC) was “humoral immune response”, “receptor-ligand activity”, and “immunoglobulin complex”, respectively.

**Figure 3:**
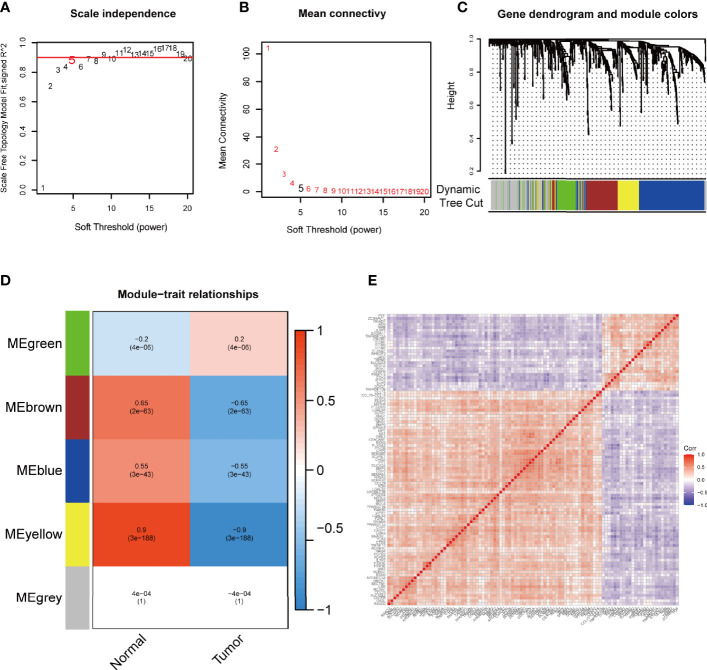
WGCNA to mine differential immune gene modules. Analysis of IRGPI-related gene modules by WGCNA. **(A)** Analysis of the scale-independence of various soft-thresholding powers. **(B)** Mean connectivity analysis of various soft-thresholding powers. **(C)** Identification of co-expression modules. The branches of the tree diagram correspond to the five different gene modules. **(D)** Correlation of gene modules with tumor microenvironment correlation scores. Each cell contains the corresponding correlation coefficient and *P*-value. **(E)** Correlation analysis of each gene in the yellow module. WGCNA, weighted gene co-expression network analysis and IRGPI, immune-related gene prognostic index.

To extract immune-related hub genes, we performed WGCNA analysis on candidate genes. According to the scale-free network, the optimal soft-thresholding power was 5 (**Figures 3A, B****)**. A dendrogram of identified co-expressed genes in modules by using 651 DEGs (**Figure 3C**). Five modules were subsequently identified in terms of the average linkage hierarchical clustering and the optimal soft-thresholding power (**Figure 3D**). According to the Pearson correlation coefficient between a module and sample feature for each module, the yellow, brown, and blue modules closely correlated with colon cancer tumors. The genes in the yellow module were selected for further analysis. The network diagram showed the significant genes, and the two nodes were connected with lines to represent co-expression relationships. The genetic correlation in the yellow module was shown in **Figure 3E**. Two main clusters of genes exhibited good consistency in this module.

### Survival Outcomes in Different IRGPI Groups

**Figure 4:**
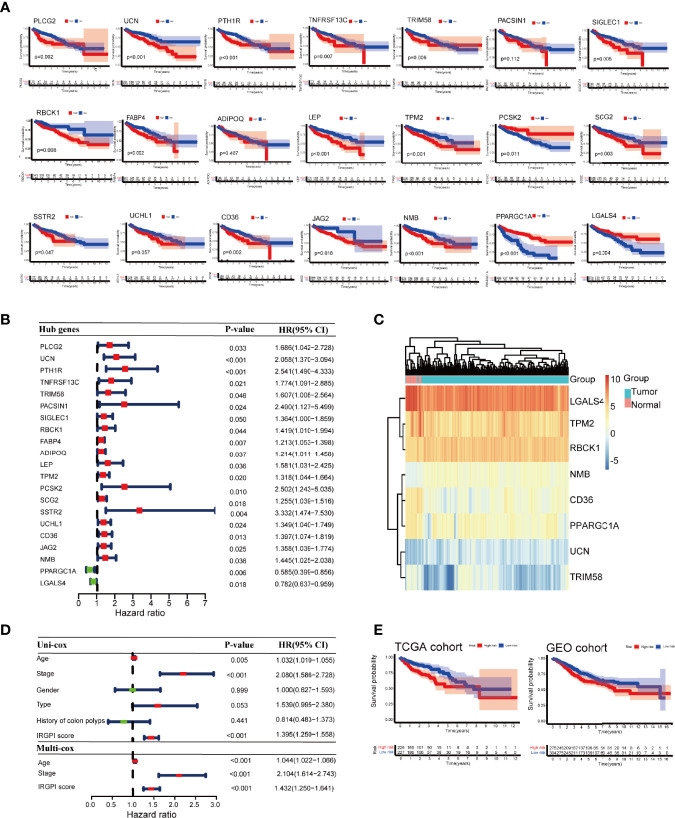
Prognostic analysis of different colon cancer groups. **(A)** Kaplan-Meier survival analysis of the 21 immune-related hub genes. **(B)** Univariate Cox analysis of 21 immune-related hub genes. **(C)** Heat map of 8 genes in IRGPI score. **(D)** Univariate Cox analysis of clinicopathological factors and IRGPI scores, and multivariate Cox analysis of factors significant in univariate Cox analysis (*P* < 0.05). **(E)** Kaplan-Meier survival analysis of IRGPI groups in the TCGA cohort and the GEO cohort. IRGPI, immune-related gene prognostic index; TCGA, The Cancer Genome Atlas; and GEO, Gene Expression Omnibus.

Kaplan-Meier survival analysis showed that the expression of 21 immune-related hub genes was strongly linked to OS in patients with colon cancer, as shown in **Figure 4A**. To select independent prognostic genes, multivariate Cox regression analysis for OS was performed among the 21 immune-related hub genes. As shown in **Figures 4A, B**, only eight genes (UCN, TRIM, RBCK1, TPM2, CD36, NMB, PPARGC1A, and LGALS4) significantly affected the OS in patients with colon cancer (*P* < 0.05). Following these results,we constructed a prognostic index for all cancer samples, with the following formula:


IRGPI=UCN∗0.775+TRIM58∗0.736+RBCK1∗0.304+TPM2∗0.241+CD36∗0.319+NMB∗0.281+PPARGC1A∗(−0.359)+LGALS4∗(−0.211)

The heatmap of the 8 genes presented well-distinguished clusters between the normal and tumor samples (**Figure 4C**). Age and stage were significantly associated with the prognosis of colon cancer as shown by univariate Cox regression analysis. Multivariate Cox regression analysis revealed that IRGPI was an independent prognostic factor, after being adjusted for other clinicopathologic factors (**Figure 4D**).

Taking the median IRGPI as the cutoff value, IRGPI-low group had a better OS than IRGPI-high group (*P* = 0.002). The role of IRGPI was then validated by the GSE39582 (n = 585) colon cancer dataset. Patients in the IRGPI-low group had a significantly better prognosis than those in the IRGPI-high group, which was consistent with the result of the TCGA dataset (*P* = 0.022; **Figure 4E**).

### Molecular Characteristics of Different IRGPI Groups

**Figure 5:**
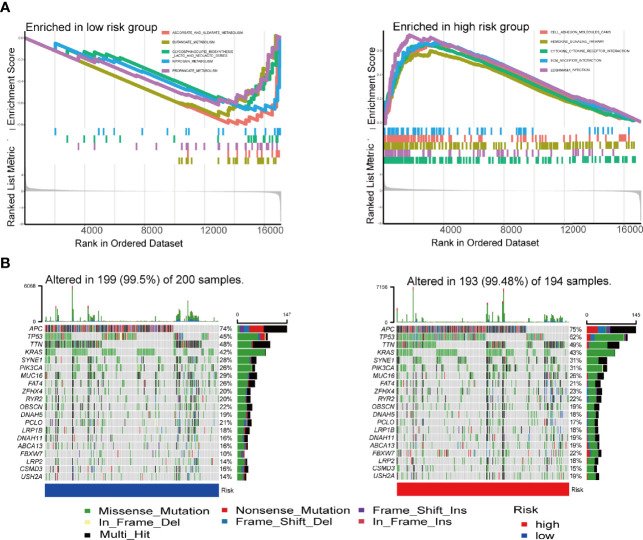
GSEA analysis and mutation analysis of IRGPI groups. **(A)** Set of genes enriched in IRGPI groups (*P* < 0.05). **(B)** Significantly mutated genes in the colon cancer samples of different IRGPI groups. Mutated genes are sorted by mutation rate; the arrangement of samples (columns) emphasizes the mutual exclusivity between mutations. Mutation rates are shown on the right, and the total number of mutations is shown above. Color coding indicates mutation type. GSEA, genomic-based gene set enrichment analysis; IRGPI, immune-related gene prognostic index.

GSEA analysis was carried out to explore the gene pathways enriched in different IRGPI groups. IRGPI-high group was enriched in cell adhesion molecules (CAMs) and chemokine signaling pathways, while IRGPI-low group was enriched in antitumor metabolisms, such as ascorbate and aldarate metabolism and butanoate metabolism (*P* < 0.05; **Figure 5A**).

Subsequently, we analyzed gene mutations to further understand the immunological nature of the IRGPI subpopulation (**Figure 5B**). To gain further biological insight into the immunological nature of the IRGPI groups. Missense variations were the most frequent mutation type. Most of the 21 immune-associated hub genes had missense mutations and frame shift mutation.In fact, mutations of APC, TTN, TP53, KRAS, and MUC16 were the 5 most prevalent mutations in the IRGPI-low group, whereas mutations of APC, TP53, TTN, KRAS, and SYNE1 were the 5 most prevalent mutations in IRGPI-high group.

### Immune Characteristics of Different IRGPI Groups

**Figure 6:**
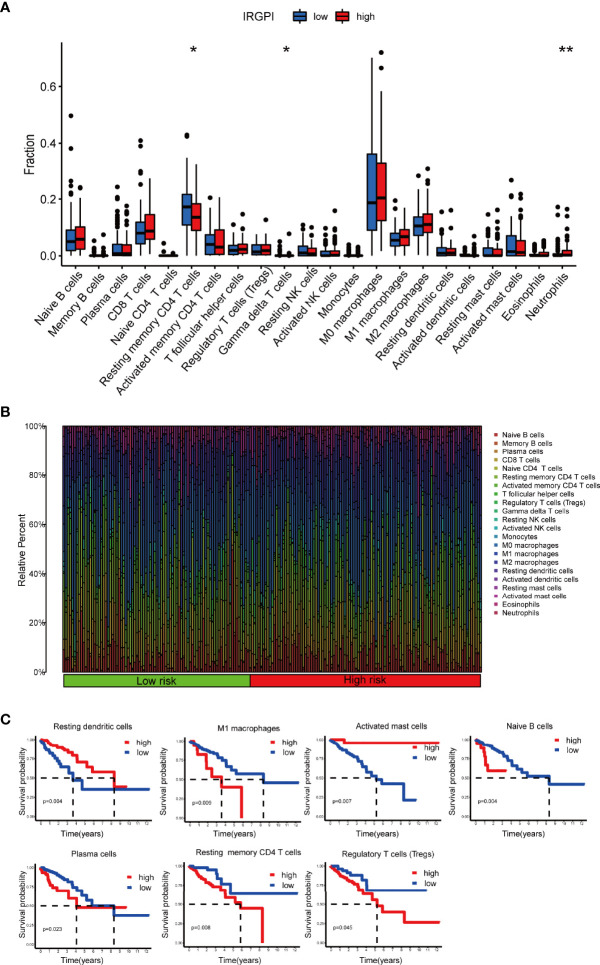
TME landscape of colon cancer and characterization of different IRGPI groups. The proportions of TME cells in the high and low IRGPI groups. **(A)** The correlation of IRPGI scores with 22 immune cells. **(B)** Characteristics associated with the immune landscape. **(C)** Kaplan-Meier survival analysis of the correlation of immune cell abundance ratios in the IRGPI group. (*P < 0.05; **P < 0.01). TME, tumor immune microenvironment; IRGPI, immune-related gene prognostic index.

The composition of immune cells in different IRGPI groups was analyzed using Wilcoxon test. The test compared the fraction of immune cells in different IRGPI groups. Here, we found that resting memory CD4 T cells and gammadelta T cells (γδ T cells) were more abundant in the IRGPI-low group, while neutrophils were more abundant in the IRGPI-high group (**Figure 6A**). The abundance ratios of 22 immune cells were displayed in **Figure 6B**. Consequently, the correlations between the abundance ratios of immune cells were analyzed using Kaplan-Meier survival analysis in overall survival. Seven immune cell types associated with survival were presented in the survival analysis performed on 22 immune cells. The relative proportion of naive B cells, plasma cells, resting memory CD4 T cells, regulatory T cells (Tregs), M1 macrophages, resting dendritic cells and activated mast cells were significantly related to OS (P < 0.05). Higher abundance ratios of M1 macrophages, naive B cells, plasma cells, resting memory CD4 T cells, and Tregs were associated with poorer OS, while a higher proportion of activated memory dendritic cells resting and activated mast cells were related to better OS (**Figure 6C**).

We then applied certain gene signatures to define the immune and molecular function between different IRGPI groups (19). We further investigated whether the prognostic value of IRGPI resulted from better immune control or less aggressive cancer growth. We found that patients with a lower score had a better outcome, with more resting memory CD4 T cells and γδ T cells infiltration. Less cytolytic activity, dendritic cells, plasmacytoid dendritic cells and plasmacytoid dendritic cells had a favorable prognosis. Therefore, we proposed that the prognostic value of IRGPI might result from both better immune control and less aggressive cancer growth. Collectively, IRGPI statistically correlated with the infiltration levels of most immune cells, implying that our IRGPI could potentially reflect the state of TME. In a nutshell, IRGPI was statistically associated with the levels of infiltration of immune cells, and immune function, which means that our IRGPI could potentially reflect the status of TME.

### Relationship Between IRGPI Groups and Other Immune and Molecular Subtypes

**Figure 7:**
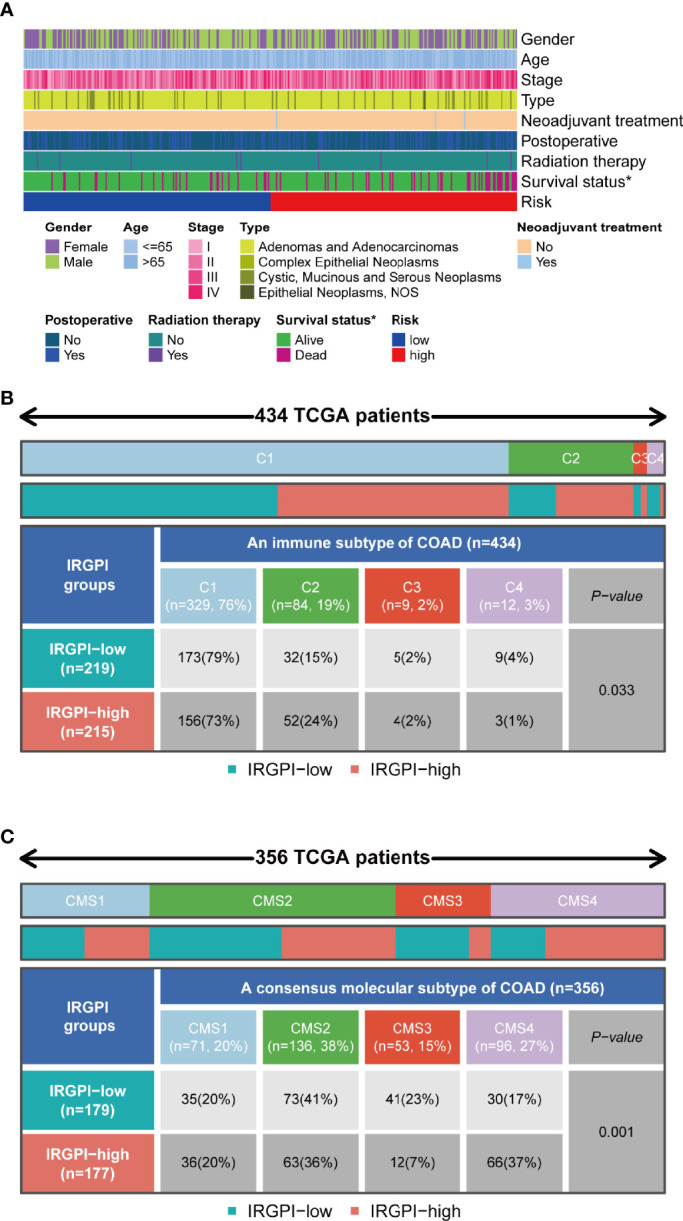
Immunological and molecular subtype distribution of different IRGPI groups. **(A)** Heat map of IRGPI grouped clinical factors for colon cancer patients. **(B)** Heat map and table showing the distribution of colon cancer immune subtypes (C1, C2, C3, and C4) between the IRGPI groups. **(C)** Heat map and table showing the distribution of molecular subtypes (CMS1, CMS2, CMS3, and CMS4) between the IRGPI groups. IRGPI, immune-related gene prognostic index; CMS, consensus molecular subtypes. *P < 0.05.

Features related to the immune landscape, including clinicopathological characteristics of different IRGPI groups, could be found in **Figure 7A**. The information about microsatellite instability (MSI) status of patients and chemotherapy were analyzed in relation to IRGPI (**Figure S1**). EGFR and KRAS status were analyzed in relation to IRGPI (**Table S1**). In conclusion, IRGPI was an independent prognostic factor for other clinicopathologic factors. Colon cancer immune subtype classification described the immune landscape according to the tumor and stromal compartments and summarized six immune subtypes: wound healing(C1), IFN-γ dominant(C2), inflammatory(C3), lymphocyte depleted(C4), immunologically quiet(C5) and TGF-β dominant(C6) (25, 26). The proportion of each group was approximately the same, with C2 being more in the high-risk group (**Figure 7B**). A unified transcriptomic classification identified four biologically distinct consensus molecular subtypes (CMSs) (27, 28): CMS1 (MSI immune subtype, 14%), characterized by BRAF mutation enriched, hypermutated and hypermethylated tumors, with a strong immune activation; CMS2(canonical subtype, 37%), commonly CIN tumors with upregulation of WNT and MYC signaling; CMS3 (metabolic subtype, 13%), encompassed epithelial tumors with metabolic deregulation, enriched in KRAS mutations; and CMS4 (mesenchymal subtype, 23%), defined by strong activation of epithelial-emesenchymal transition, angiogenesis, and stemness pathways (28). CMS4 was the subtype with the worst outcome (27).

As shown in **Figure 7B**, the IRGPI-low group included 79% of C1, 15% of C2, 2% of C3, and 4% of C4 samples in our study, whereas the group with high IRGPI score included 73% of C1, 24% of C2, 2% of C3, and 1% of C4 samples (*P* = 0.033). As shown in **Figure 7C**, the IRGPI-low group comprised 20% of CMS1, 41% of CMS2, 23% of CMS3, and 17% of CMS4 samples, while the IRGPI-high group comprised 20% of CMS1, 36% of CMS2, 7% of CMS3, and 37% of CMS4 samples. There were more CMS2 and CMS3 samples in the IRGPI-low group, while more CMS4 subtypes in the IRGPI-high group (*P* = 0.001).

### Benefits of ICI Treatment in Different IRGPI Groups

**Figure 8:**
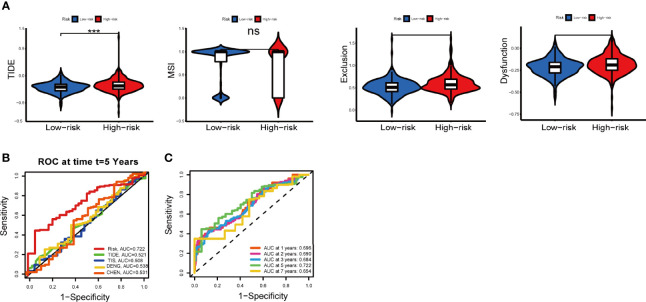
Immune response to ICI therapy and the prognostic value. **(A)** TIDE, MSI, and T cell exclusion and dysfunction score for different IRGPI groups. Scores were compared between the two IRGPI groups (ns, not significant; ***P < 0.001). **(B)** Kaplan-Meier survival analysis of the IRGPI groups in TIDE, TIS, the colon cancer cohorts of Deng et al. and Chen et al. **(C)** Time-dependent ROC curve analysis. ICI, immune checkpoint inhibitor; MSI, microsatellite instability; TIS, T cell-inflamed signature; IRGPI, immune-related gene prognostic index; ROC, receiver operating characteristic.

The potential efficacy ICI treatment in different IRGPI groups can be assessed with TIDE algorithm. A higher TIDE score indicated higher potential for immune evasion, meaning that patients were less likely to benefit from ICI treatment (29). In our results, the IRGPI-low group had a lower TIDE score than the IRGPI-high group, implying that IRGPI-low patients could benefit more from ICI therapy than IRGPI-high patients (**Figure 8A**). Furthermore, a higher TIDE score prediction score was associated with a worse outcome. Therefore, the IRGPI-low group with a low TIDE score might had a better outcome than the IRGPI-high group with a high TIDE score. We also found no difference in microsatellite instability (MSI) score between the two groups. The IRGPI-low group had a lower T cell exclusion score and T cell dysfunction compared with the IRGPI-high group.

In addition, the predictive performance of IRGPI was compared with other signatures through ROC curves. Here, the AUC at five-year of IRGPI was 0.722, compared with widely used biomarkers and other published immune-related features for cancer immunotherapy, achieving superior performance. (**Figure 8B**). The AUC was 0.696, 0.690, 0.684, 0.722 and 0.654 for 1-year, 2-year, 3-year, 5-year, and 7-year survival, respectively (**Figure 8C**). Overall, these results indicated that IRGPI was a highly reliable index and superior to other signatures.

## Discussion

ICI therapy is an effective treatment for patients with colon cancer (30). Because the overall response rate to ICI therapy remains very low (31), it is critical to identify patients who can benefit the most from these treatments. Complex cellular and molecular interactions between the tumor and host immune system influence tumor progression and patient survival (32). After years of evaluating different prognostic markers in colon cancer, we still have not found validated biomarkers for predicting response to immunotherapy and OS. This highlights the need to identify prognostic biomarkers for use in immunotherapy in colon cancer.

There is growing evidence that cancer evolution is strongly dependent on the complex TME. The main immune parameters associated with survival are defined as the “immune environment” (32, 33), which is the type, functional localization, density, and location of adaptive immune cells in different tumor areas (32–35). Several soluble factors such as cytokines, chemokines, and cellular metabolites are also included (36). WGCNA can correlate modules with specific clinical features, from which genes performing key functions can be identified. In our study, based on the colon cancer immune gene datasets, we used WGCNA to identify 21 immune-related hub genes affecting patients’ OS and constructed IRGPI based on 8 independent OS prognostic factors (UCN, TRIM58, RBCK1, TPM2, CD36, NMB, PPARGC1A, and LGALS4). IRGPI is a valid biomarker for predicting the prognosis of colon cancer. Patients with low IRGPI score had better survival, and patients with high IRGPI score had poorer survival in the TCGA and GEO cohorts.

IRGPI was composed of 8 genes UCN, TRIM58, RBCK1, TPM2, CD36, NMB, PPARGC1A, and LGALS4. Urocortins (UCNs) are adrenergic releasing factor-related peptides that regulate gastrointestinal motility and visceral pain in response to stress (37). UCNs can inhibit the growth of colon cancer tumor cells by inducing apoptosis through PUMA and p53 targets (38). TRIM58 expression was markedly inhibited in CRC and negatively related to CRC progression (39). Hypermethylation of TRIM58 down-regulation of its mRNA expression correlates with prognosis of various digestive tract tumors, suggesting that high expression is linked to bad outcome (40). RBCK1 is associated with the sensitivity and stemness of chemotherapeutic drugs in CRC (41). TPM2 (β -Tropomyosin), encoded tropomyosin β chain, was identified as a fibroblast-specific biomarker of poor prognosis in CRC (42). CD36 regulates cell-attachment-to-extracellular matrix attachment, stromal cell fate, TGFβ activation, and immune signaling, which is an early marker of cancer invasion and metastasis in breast, prostate, ovarian, liver, and colon cancer (43). PPARGC1A has an essential function in the modulation of mitochondrial biogenesis and metabolism and protects against tumorigenesis by regulating the fate enterocytes (44, 45). LGALS4 inhibits tumor cell infiltration (46), and LGALS4 upregulation prolongs disease-free survival in CRC (47). Detailed links between these genes are yet to be elucidated. In addition, more research is warranted to uncover the mechanism of action of these genes in colon cancer.

To further understand the immunological nature of IRGPI groups, we investigated gene mutations in different IRGPI groups. The mutation landscape showed that APC and TP53 gene mutation were more frequent in both high and low risk groups, and the mutation rates were greater in the IRGPI-high group than in the IRGPI-low group for APC (74% vs 45%) and TP53 (62% vs 45%). Mutations in the tumor suppressor gene APC, found in approximately 80% of human colon cancer, disrupt intestinal stem cell (ISC) homeostasis and lead to unrestricted activation of the WNT pathway (48). TP53 mutation leads to loss of function, genome-wide ploidy, and local oncogene amplification in the colon (49). There is growing evidence that TP53 mutations affect the TME (50). Specific mutant p53 proteins are capable of producing non-cell-autonomous effects, such as secreting higher levels of interferon-β (51), inducing paracrine effects, mediating the M1 polarization pattern in neighboring macrophages (52), and reprogramming neighboring macrophages (53). Thus, patients in the IRGPI-high group with high APC and TP53 mutations have a worse prognosis than patients in the IRGPI-low group with low APC and TP53 mutations, which is consistent with our survival results.

Considering the importance of immune cells in tumor immune infiltration, we further evaluated the relative proportion of 22 immune cells in each colon cancer specimen and assessed the immune function. Some evidence suggests that the interaction between tumor and microenvironment plays a crucial role in the progression of colon cancer and the probability of response to immunotherapy. Previous studies found an association between reduced resting memory CD4 T cell infiltration and poor prognosis (54). Indeed, γδ T cells are a subset of cytotoxic T cells that produce TNF-α, leading to tumor elimination through their powerful effector function. They can recruit other immune cells and increase the high antitumor activity in colon cancer. In addition, neutrophils play a key role in tumorigenesis and tumor promotion and metastasis by increasing angiogenesis, cell motility, migration, and invasion. Neutrophils can amplify DNA damage in cancer cells through reactive oxygen species(ROC), promoting liver metastasis. M1 macrophages have pro-inflammatory and tumoricidal properties (55). However, the prognostic significance of macrophages in the microenvironment of CRC is not fully understood. Several studies have assessed the prognostic significance of macrophages in CRC, but the results are conflicting (56–65). Colon cancer progression is usually associated with a systemic inflammatory response, and multiple mechanisms of inflammatory mediators support tumor growth and spread (66, 67).Different tumor-associated macrophages (TAM) subpopulations have opposing functions and specific infiltration sites, hence the role of TAM in the prognosis of colon cancer has not been fully defined (68). Furthermore, IRGPI-low samples were enriched for stronger anti-tumor metabolism and inhibition of tumor metastasis, while IRGPI-high samples had more immunosuppressive cells and signals associated with tumor and metastasis, suggesting that the IRGPI-high group was characterized by immunosuppression and active tumor progression.

IRGPI groups could distinguish different molecular and immunological subtypes of colon cancer. Thorsson et al. developed a new worldwide immune classification of solid tumors from transcriptional profiles of more than 10,000 patients from all TCGAs (69). According to their classification, colon cancer immune subtypes were mainly composed of C1, C2, C3, and C4. Among them, colon cancer was dominated by C1, with increased expression of angiogenic genes associated with adaptive immune infiltration. In C1, the proportion of the IRGPI-low group was higher than the IRGPI-high group, suggesting that IRGPI was associated with adaptive immune infiltration. The International CRC Subtype Consortium has developed a unified transcript classification based on four biologically distinct CMS, which is widely accepted (27): CMS1, CMS2, CMS3, and CMS4. Among them, CMS2 was activated with WNT and MYC signaling with an intermediate prognosis. CMS3 epithelial cells had a marked metabolic disorder with an intermediate prognosis. CMS4 had prominent TNF-β activation and a poor prognosis. In our study, the proportion of CMS2 and CMS3 was higher in the IRGPI-low group than IRGPI-high group, respectively. Conversely, CMS4 was lower in the IRGPI-low group than IRGPI-high group. It was clarified that patients with low IRGPI scores had active immunity and a good prognosis, whereas patients with high IRGPI scores had immunosuppressive features and a poor prognosis, which was consistent with our previous findings.

To Assess the response to ICI therapy in patients with colon cancer, we forecasted the probability of the IRGPI model using TIDE and TIS scores. The TIDE score was a newly developed immunotherapy response prediction method for forecasting the effectiveness of anti-PD1 and anti-CTLA4 therapy more accurately than TMB or PD-L1 expression (70). A higher TIDE score indicated poorer tumor response to ICI therapy and a worse prognosis (20). In our study, TIDE scores were higher in the IRGPI-high group than the IRGPI-low group, suggesting a large immune escape and poor outcome with high IRGPI scores. This suggested that IRGPI was a valid biomarker for predicting response to immunotherapy. The low IRGPI score group may be more sensitive to immunotherapy. As for TIS score, it was described as a biomarker for predicting prognosis of patients with different types of cancer (21). The clinical trial assay, running on the nCounter Analysis System, contained genes associated with antigen presentation, chemokine expression, cytotoxic activity, and adaptive immune resistance (71). Our IRGPI score was more sensitive compared to the TIDE and TIS scores, and hence better. In addition, we compared IRGPI score with Zhang’s signature and Deng’s signature in terms of predicting immune prognosis and consistently found that IRGPI score had a higher sensitivity. In conclusion, our study developed a reliable immune-related risk signature that could predict survival and response to ICI therapy in patients with colon cancer. To our knowledge, this is the first IRG prognostic model based on WGCNA to differentiate the prognostic, molecular, and immunological profile for colon cancer. It played an important role in differentiating immune and molecular features and predicting patient prognosis. However, the study still has some limitations. First, the data were based on retrospective datasets, and a prospective study of this IRGPI-based signature would be necessary. Second, all expression data were sequencing data downloaded from public databases, and the results need to be validated by new methods and external experiments with fresh specimens. Finally, indirect assessment of IRGPI’s ability to predict response to immunotherapy would require further studies in large-scale multicenter studies.

## Data Availability Statement

The datasets presented in this study can be found in online repositories. The names of the repository/repositories and accession number(s) can be found in the article/**Supplementary Material**.

## Author Contributions

ZW and JS contributed to the methodology and formal analysis. JS and ZW contributed to write the manuscript. NA polished English writing of the manuscript. MS contributed to the funding, writing, review, and editing. All authors contributed to the article and approved the submitted version.

## Funding

This work was supported by the major project of Shanghai Municipal S and T Commission (no. 19401972300), Shandong Province Key R&D Program (Major Science and Technology Innovation Project, 2021CXGC010509), Shanghai Key Laboratory of Traditional Chinese Clinical Medicine, Key Disciplines of Liver and Gall Bladder Diseases, and Key Laboratory of Chronic Deficiency Liver Disease of the State Administration of Traditional Chinese Medicine of the People’s Republic of China (20DZ2272200). Shanghai University of Traditional Chinese Medicine Postgraduate Innovation Training Program (Y2021087).

## Conflict of Interest

The authors declare that the research was conducted in the absence of any commercial or financial relationships that could be construed as a potential conflict of interest.

## Publisher’s Note

All claims expressed in this article are solely those of the authors and do not necessarily represent those of their affiliated organizations, or those of the publisher, the editors and the reviewers. Any product that may be evaluated in this article, or claim that may be made by its manufacturer, is not guaranteed or endorsed by the publisher.

## Supplementary Material

Supplementary Figure 1 Relationship between MSI status, chemotherapy in IRGPI groups.**(A)** MSI status. **(B)** Chemotherapy. MSI, microsatellite instability.

## References

[1] Arnold M Sierra M Laversanne M Soerjomataram I Jemal A Bray FJG. Global Patterns and Trends in Colorectal Cancer Incidence and Mortality. Gut (2017) 66(4):683–91. doi: 10.1136/gutjnl-2015-310912 26818619

[2] Labianca R Nordlinger B Beretta GD Mosconi S Mandalà M Cervantes A. Early Colon Cancer: Esmo Clinical Practice Guidelines for Diagnosis, Treatment and Follow-Up. Ann Oncol (2013) (24 Suppl 6):vi64–72. doi: 10.1093/annonc/mdt354 24078664

[3] Zhang T Xie J Arai S Wang L Shi X Shi N. The Efficacy and Safety of Anti-Pd-1/Pd-L1 Antibodies for Treatment of Advanced or Refractory Cancers: A Meta-Analysis. Oncotarget (2016) 7(45):73068–79. doi: 10.18632/oncotarget.12230 PMC5341964 27683031

[4] D'Angelo SP Larkin J Sosman JA Lebbé C Brady B Neyns B. Efficacy and Safety of Nivolumab Alone or in Combination With Ipilimumab in Patients With Mucosal Melanoma: A Pooled Analysis. J Clin Oncol Off J Am Soc Clin Oncol (2017) 35(2):226–35. doi: 10.1200/jco.2016.67.9258 PMC5559888 28056206

[5] Binnewies M Roberts EW Kersten K Chan V Fearon DF Merad M. Understanding the Tumor Immune Microenvironment (Time) for Effective Therapy. Nat Med (2018) 24(5):541–50. doi: 10.1038/s41591-018-0014-x PMC5998822 29686425

[6] Nishino M Ramaiya NH Hatabu H Hodi FS. Monitoring Immune-Checkpoint Blockade: Response Evaluation and Biomarker Development. Nat Rev Clin Oncol (2017) 14(11):655–68. doi: 10.1038/nrclinonc.2017.88 PMC5650537 28653677

[7] Taube JM Galon J Sholl LM Rodig SJ Cottrell TR Giraldo NA. Implications of the Tumor Immune Microenvironment for Staging and Therapeutics. Mod Pathol an Off J United States Can Acad Pathol Inc (2018) 31(2):214–34. doi: 10.1038/modpathol.2017.156 PMC6132263 29192647

[8] Marisa L de Reyniès A Duval A Selves J Gaub M Vescovo L. Gene Expression Classification of Colon Cancer Into Molecular Subtypes: Characterization, Validation, and Prognostic Value. PloS Med (2013) 10(5):e1001453. doi: 10.1371/journal.pmed.1001453 23700391 PMC3660251

[9] Ritchie M Phipson B Wu D Hu Y Law C Shi W. Limma Powers Differential Expression Analyses for Rna-Sequencing and Microarray Studies. Nucleic Acids Res (2015) 43(7):e47. doi: 10.1093/nar/gkv007 25605792 PMC4402510

[10] Yu G Wang LG Han Y He QY. Clusterprofiler: An R Package for Comparing Biological Themes Among Gene Clusters. Omics J Integr Biol (2012) 16(5):284–7. doi: 10.1089/omi.2011.0118 PMC3339379 22455463

[11] Zhang B Horvath S. A General Framework for Weighted Gene Co-Expression Network Analysis. Stat Appl Genet Mol Biol (2005) 4:Article17. doi: 10.2202/1544-6115.1128 16646834

[12] Niemira M Collin F Szalkowska A Bielska A Chwialkowska K Reszec J. Molecular Signature of Subtypes of Non-Small-Cell Lung Cancer by Large-Scale Transcriptional Profiling: Identification of Key Modules and Genes by Weighted Gene Co-Expression Network Analysis (Wgcna). Cancers (2019) 12(1):37. doi: 10.3390/cancers12010037 PMC7017323 31877723

[13] Lorent M Giral M Foucher Y. Net Time-Dependent Roc Curves: A Solution for Evaluating the Accuracy of a Marker to Predict Disease-Related Mortality. Stat Med (2014) 33(14):2379–89. doi: 10.1002/sim.6079 24399671

[14] Mayakonda A Lin DC Assenov Y Plass C Koeffler HP. Maftools: Efficient and Comprehensive Analysis of Somatic Variants in Cancer. Genome Res (2018) 28(11):1747–56. doi: 10.1101/gr.239244.118 PMC6211645 30341162

[15] He Y Jiang Z Chen C Wang X. Classification of Triple-Negative Breast Cancers Based on Immunogenomic Profiling. J Exp Clin Cancer Res CR (2018) 37(1):327. doi: 10.1186/s13046-018-1002-1 30594216 PMC6310928

[16] Zeng D Ye Z Wu J Zhou R Fan X Wang G. Macrophage Correlates With Immunophenotype and Predicts Anti-Pd-L1 Response of Urothelial Cancer. Theranostics (2020) 10(15):7002–14. doi: 10.7150/thno.46176 PMC7295060 32550918

[17] Hwang S Kwon AY Jeong JY Kim S Kang H Park J. Immune Gene Signatures for Predicting Durable Clinical Benefit of Anti-Pd-1 Immunotherapy in Patients With Non-Small Cell Lung Cancer. Sci Rep (2020) 10(1):643. doi: 10.1038/s41598-019-57218-9 31959763 PMC6971301

[18] Tirosh I Izar B Prakadan SM Wadsworth MH 2nd Treacy D Trombetta JJ. Dissecting the Multicellular Ecosystem of Metastatic Melanoma by Single-Cell Rna-Seq. Sci (New York NY) (2016) 352(6282):189–96. doi: 10.1126/science.aad0501 PMC4944528 27124452

[19] Blanche P Dartigues JF Jacqmin-Gadda H. Estimating and Comparing Time-Dependent Areas Under Receiver Operating Characteristic Curves for Censored Event Times With Competing Risks. Stat Med (2013) 32(30):5381–97. doi: 10.1002/sim.5958 24027076

[20] Jiang P Gu S Pan D Fu J Sahu A Hu X. Signatures of T Cell Dysfunction and Exclusion Predict Cancer Immunotherapy Response. Nat Med (2018) 24(10):1550–8. doi: 10.1038/s41591-018-0136-1 PMC6487502 30127393

[21] Ayers M Lunceford J Nebozhyn M Murphy E Loboda A Kaufman D. Ifn-Γ-Related Mrna Profile Predicts Clinical Response to Pd-1 Blockade. J Clin Invest (2017) 127(8):2930–40. doi: 10.1172/jci91190 PMC5531419 28650338

[22] Damotte D Warren S Arrondeau J Boudou-Rouquette P Mansuet-Lupo A Biton J. The Tumor Inflammation Signature (Tis) Is Associated With Anti-Pd-1 Treatment Benefit in the Certim Pan-Cancer Cohort. J Trans Med (2019) 17(1):357. doi: 10.1186/s12967-019-2100-3 PMC6829827 31684954

[23] Chen W Huang J Xiong J Fu P Chen C Liu Y. Identification of a Tumor Microenvironment-Related Gene Signature Indicative of Disease Prognosis and Treatment Response in Colon Cancer. Oxid Med Cell Longev (2021) 2021:6290261. doi: 10.1155/2021/6290261 34497681 PMC8420973

[24] Deng D Luo X Zhang S Xu ZJA. Immune Cell Infiltration-Associated Signature in Colon Cancer and Its Prognostic Implications. Aging (Albany NY) (2021) 13(15):19696–709. doi: 10.18632/aging.203380 PMC8386549 34349038

[25] Thorsson V Gibbs DL Brown SD Wolf D Bortone DS Ou Yang T-H. The Immune Landscape of Cancer. Immunity (2018). 48(4):812–30. doi: 10.1016/j.immuni.2018.03.023 PMC5982584 29628290

[26] Xu Y Li D Liu Z Gibbs DL Xie L Qin G. Intrinsic Genetic and Transcriptomic Patterns Reflect Tumor Immune Subtypes Facilitating Exploring Possible Combinatory Therapy. Front Mol Biosci (2020) 7:53. doi: 10.3389/fmolb.2020.00053 32391377 PMC7191006

[27] Guinney J Dienstmann R Wang X de Reyniès A Schlicker A Soneson C. The Consensus Molecular Subtypes of Colorectal Cancer. Nat Med (2015) 21(11):1350–6. doi: 10.1038/nm.3967 PMC4636487 26457759

[28] Soldevilla B Carretero-Puche C Gomez-Lopez G Al-Shahrour F Riesco MC Gil-Calderon B. The Correlation Between Immune Subtypes and Consensus Molecular Subtypes in Colorectal Cancer Identifies Novel Tumour Microenvironment Profiles, With Prognostic and Therapeutic Implications. Eur J Cancer (Oxford Engl 1990) (2019) 123:118–29. doi: 10.1016/j.ejca.2019.09.008 31678770

[29] Li D Lin X Chen B Ma Z Zeng Y Wang H. Identification and Validation of Emt-Related Lncrna Prognostic Signature for Colorectal Cancer. Front Genet (2021) 12:723802. doi: 10.3389/fgene.2021.723802 34659346 PMC8513715

[30] Lichtenstern CR Ngu RK Shalapour S Karin M. Immunotherapy, Inflammation and Colorectal Cancer. Cells (2020) 9(3):618. doi: 10.3390/cells9030618 PMC7140520 32143413

[31] Xiao W Ibrahim ML Redd PS Klement JD Lu C Yang D. Loss of Fas Expression and Function Is Coupled With Colon Cancer Resistance to Immune Checkpoint Inhibitor Immunotherapy. Mol Cancer Res MCR (2019) 17(2):420–30. doi: 10.1158/1541-7786.Mcr-18-0455 PMC6359951 30429213

[32] Galon J Angell HK Bedognetti D Marincola FM. The Continuum of Cancer Immunosurveillance: Prognostic, Predictive, and Mechanistic Signatures. Immunity (2013) 39(1):11–26. doi: 10.1016/j.immuni.2013.07.008 23890060

[33] Galon J Fridman W Pagès F. The Adaptive Immunologic Microenvironment in Colorectal Cancer: A Novel Perspective. Cancer Res (2007) 67(5):1883–6. doi: 10.1158/0008-5472.Can-06-4806 17332313

[34] Angell H Galon J. From the Immune Contexture to the Immunoscore: The Role of Prognostic and Predictive Immune Markers in Cancer. Curr Opin Immunol (2013) 25(2):261–7. doi: 10.1016/j.coi.2013.03.004 23579076

[35] Fridman WH Pagès F Sautès-Fridman C Galon J. The Immune Contexture in Human Tumours: Impact on Clinical Outcome. Nat Rev Cancer (2012) 12(4):298–306. doi: 10.1038/nrc3245 22419253

[36] Bao X Shi R Zhao T Wang Y. Mast Cell-Based Molecular Subtypes and Signature Associated With Clinical Outcome in Early-Stage Lung Adenocarcinoma. Mol Oncol (2020) 14(5):917–32. doi: 10.1002/1878-0261.12670 PMC7191192 32175651

[37] Martinez V Wang L Million M Rivier J Taché YJP. Urocortins and the Regulation of Gastrointestinal Motor Function and Visceral Pain. Peptides (2004) 25(10):1733–44. doi: 10.1016/j.peptides.2004.05.025 15476940

[38] Dudgeon C Wang P Sun X Peng R Sun Q Yu J. Puma Induction by Foxo3a Mediates the Anticancer Activities of the Broad-Range Kinase Inhibitor Ucn-01. Mol Cancer Ther (2010) 9(11):2893–902. doi: 10.1158/1535-7163.Mct-10-0635 PMC2978764 20978166

[39] Liu M Zhang X Cai J Li Y Luo Q Wu H. Downregulation of Trim58 Expression Is Associated With a Poor Patient Outcome and Enhances Colorectal Cancer Cell Invasion. Oncol Rep (2018) 40(3):1251–60. doi: 10.3892/or.2018.6525 PMC6072390 29956813

[40] Qiu X Huang Y Zhou Y Zheng F. Aberrant Methylation of Trim58 in Hepatocellular Carcinoma and Its Potential Clinical Implication. Oncol Rep (2016) 36(2):811–8. doi: 10.3892/or.2016.4871 27373520

[41] Liu M Zang F Zhang SJB. Biomedecine P, Pharmacotherapie. Rbck1 Contributes to Chemoresistance and Stemness in Colorectal Cancer (Crc). BioMed Pharmacother (2019) 118:109250. doi: 10.1016/j.biopha.2019.109250 31545242

[42] Zhou Y Bian S Zhou X Cui Y Wang W Wen L. Single-Cell Multiomics Sequencing Reveals Prevalent Genomic Alterations in Tumor Stromal Cells of Human Colorectal Cancer. Cancer Cell (2020) 38(6):818–28.e5. doi: 10.1016/j.ccell.2020.09.015 33096021

[43] Enciu AM Radu E Popescu ID Hinescu ME Ceafalan LC. Targeting Cd36 as Biomarker for Metastasis Prognostic: How Far From Translation Into Clinical Practice? BioMed Res Int (2018) 2018:7801202. doi: 10.1155/2018/7801202 30069479 PMC6057354

[44] D'Errico I Salvatore L Murzilli S Lo Sasso G Latorre D Martelli N. Peroxisome Proliferator-Activated Receptor-Gamma Coactivator 1-Alpha (Pgc1alpha) Is a Metabolic Regulator of Intestinal Epithelial Cell Fate. Proc Natl Acad Sci United States America (2011) 108(16):6603–8. doi: 10.1073/pnas.1016354108 PMC3081029 21467224

[45] Cho YA Lee J Oh JH Chang HJ Sohn DK Shin A. Genetic Variation in Ppargc1a May Affect the Role of Diet-Associated Inflammation in Colorectal Carcinogenesis. Oncotarget (2017) 8(5):8550–8. doi: 10.18632/oncotarget.14347 PMC5352421 28051997

[46] Long M Campbell MJT. Integrative Genomic Approaches to Dissect Clinically-Significant Relationships Between the Vdr Cistrome and Gene Expression in Primary Colon Cancer. J Steroid Biochem Mol Biol (2017) 173:130–8. doi: 10.1016/j.jsbmb.2016.12.013. Josb, Biology M. 28027912

[47] Long MD Campbell MJ. Integrative Genomic Approaches to Dissect Clinically-Significant Relationships Between the Vdr Cistrome and Gene Expression in Primary Colon Cancer. J Steroid Biochem Mol Biol (2017) 173:130–8. doi: 10.1016/j.jsbmb.2016.12.013 28027912

[48] van Neerven S de Groot N Nijman L Scicluna B van Driel M Lecca M. Apc-Mutant Cells Act as Supercompetitors in Intestinal Tumour Initiation. Nature (2021) 594(7863):436–41. doi: 10.1038/s41586-021-03558-4 34079128

[49] Kim JE Choi J Sung CO Hong YS Kim SY Lee H. High Prevalence of Tp53 Loss and Whole-Genome Doubling in Early-Onset Colorectal Cancer. Exp Mol Med (2021) 53(3):446–56. doi: 10.1038/s12276-021-00583-1 PMC8080557 33753878

[50] Muller PA Vousden KH. Mutant P53 in Cancer: New Functions and Therapeutic Opportunities. Cancer Cell (2014) 25(3):304–17. doi: 10.1016/j.ccr.2014.01.021 PMC3970583 24651012

[51] Madar S Harel E Goldstein I Stein Y Kogan-Sakin I Kamer I. Mutant P53 Attenuates the Anti-Tumorigenic Activity of Fibroblasts-Secreted Interferon Beta. PloS One (2013) 8(4):e61353. doi: 10.1371/journal.pone.0061353 23630584 PMC3632588

[52] Trivedi M Talekar M Shah P Ouyang Q Amiji M. Modification of Tumor Cell Exosome Content by Transfection With Wt-P53 and Microrna-125b Expressing Plasmid DNA and Its Effect on Macrophage Polarization. Oncogenesis (2016) 5(8):e250. doi: 10.1038/oncsis.2016.52 27500388 PMC5007827

[53] Cooks T Pateras IS Jenkins LM Patel KM Robles AI Morris J. Mutant P53 Cancers Reprogram Macrophages to Tumor Supporting Macrophages *Via* Exosomal Mir-1246. Nat Commun (2018) 9(1):771. doi: 10.1038/s41467-018-03224-w 29472616 PMC5823939

[54] Song J Wu L. Friend or Foe: Prognostic and Immunotherapy Roles of Btla in Colorectal Cancer. Front Mol Biosci (2020) 7:148. doi: 10.3389/fmolb.2020.00148 32793631 PMC7385242

[55] López-Janeiro Á Padilla-Ansala C de Andrea CE Hardisson D Melero I. Prognostic Value of Macrophage Polarization Markers in Epithelial Neoplasms and Melanoma. A Syst Rev Meta-Analysis. Mod Pathol (2020) 33(8):1458–65. doi: 10.1038/s41379-020-0534-z 32291396

[56] Herrera M Herrera A Domínguez G Silva J García V García JM. Cancer-Associated Fibroblast and M2 Macrophage Markers Together Predict Outcome in Colorectal Cancer Patients. Cancer Sci (2013) 104(4):437–44. doi: 10.1111/cas.12096 PMC7657228 23298232

[57] Feng Q Chang W Mao Y He G Zheng P Tang W. Tumor-Associated Macrophages as Prognostic and Predictive Biomarkers for Postoperative Adjuvant Chemotherapy in Patients With Stage Ii Colon Cancer. Clin Cancer Res (2019) 25(13):3896–907. doi: 10.1158/1078-0432.Ccr-18-2076 30988081

[58] Kim Y Wen X Bae JM Kim JH Cho NY Kang GH. The Distribution of Intratumoral Macrophages Correlates With Molecular Phenotypes and Impacts Prognosis in Colorectal Carcinoma. Histopathology (2018) 73(4):663–71. doi: 10.1111/his.13674 29906313

[59] Edin S Wikberg ML Dahlin AM Rutegård J Öberg Å Oldenborg PA. The Distribution of Macrophages With a M1 or M2 Phenotype in Relation to Prognosis and the Molecular Characteristics of Colorectal Cancer. PloS One (2012) 7(10):e47045. doi: 10.1371/journal.pone.0047045 23077543 PMC3471949

[60] Forssell J Oberg A Henriksson ML Stenling R Jung A Palmqvist R. High Macrophage Infiltration Along the Tumor Front Correlates With Improved Survival in Colon Cancer. Clin Cancer Res (2007) 13(5):1472–9. doi: 10.1158/1078-0432.Ccr-06-2073 17332291

[61] Koelzer VH Canonica K Dawson H Sokol L Karamitopoulou-Diamantis E Lugli A. Phenotyping of Tumor-Associated Macrophages in Colorectal Cancer: Impact on Single Cell Invasion (Tumor Budding) and Clinicopathological Outcome. Oncoimmunology (2016) 5(4):e1106677. doi: 10.1080/2162402x.2015.1106677 27141391 PMC4839334

[62] Chaput N Svrcek M Aupérin A Locher C Drusch F Malka D. Tumour-Infiltrating Cd68+ and Cd57+ Cells Predict Patient Outcome in Stage Ii-Iii Colorectal Cancer. Br J Cancer (2013) 109(4):1013–22. doi: 10.1038/bjc.2013.362 PMC3749560 23868006

[63] Algars A Irjala H Vaittinen S Huhtinen H Sundström J Salmi M. Type and Location of Tumor-Infiltrating Macrophages and Lymphatic Vessels Predict Survival of Colorectal Cancer Patients. Int J Cancer (2012) 131(4):864–73. doi: 10.1002/ijc.26457 21952788

[64] Väyrynen JP Haruki K Lau MC Väyrynen SA Zhong R Dias Costa A. The Prognostic Role of Macrophage Polarization in the Colorectal Cancer Microenvironment. Cancer Immunol Res (2021) 9(1):8–19. doi: 10.1158/2326-6066.Cir-20-0527 33023967 PMC7785652

[65] Zhang QW Liu L Gong CY Shi HS Zeng YH Wang XZ. Prognostic Significance of Tumor-Associated Macrophages in Solid Tumor: A Meta-Analysis of the Literature. PloS One (2012) 7(12):e50946. doi: 10.1371/journal.pone.0050946 23284651 PMC3532403

[66] Tuomisto AE Mäkinen MJ Väyrynen JP. Systemic Inflammation in Colorectal Cancer: Underlying Factors, Effects, and Prognostic Significance. World J Gastroenterol (2019) 25(31):4383–404. doi: 10.3748/wjg.v25.i31.4383 PMC6710177 31496619

[67] Dolan RD Lim J McSorley ST Horgan PG McMillan DC. The Role of the Systemic Inflammatory Response in Predicting Outcomes in Patients With Operable Cancer: Systematic Review and Meta-Analysis. Sci Rep (2017) 7(1):16717. doi: 10.1038/s41598-017-16955-5 29196718 PMC5711862

[68] Li J Li L Li Y Long Y Zhao Q Ouyang Y. Tumor-Associated Macrophage Infiltration and Prognosis in Colorectal Cancer: Systematic Review and Meta-Analysis. Int J colorectal Dis (2020) 35(7):1203–10. doi: 10.1007/s00384-020-03593-z 32303831

[69] Huang TX Fu L. The Immune Landscape of Esophageal Cancer. Cancer Commun (London England) (2019) 39(1):79. doi: 10.1186/s40880-019-0427-z PMC6878621 31771653

[70] Becht E Giraldo NA Lacroix L Buttard B Elarouci N Petitprez F. Estimating The Population Abundance of Tissue-Infiltrating Immune and Stromal Cell Populations Using Gene Expression. Genome Biol (2016) 17(1):218. doi: 10.1186/s13059-016-1070-5 27765066 PMC5073889

[71] Danaher P Warren S Lu R Samayoa J Sullivan A Pekker I. Pan-Cancer Adaptive Immune Resistance as Defined by the Tumor Inflammation Signature (Tis): Results From the Cancer Genome Atlas (Tcga). J immunother Cancer (2018) 6(1):63. doi: 10.1186/s40425-018-0367-1 29929551 PMC6013904

