# Supplementary figures and images for: Identification of a Novel Immune Landscape Signature for Predicting Prognosis and Response of Colon Cancer to Immunotherapy
Source: Front Immunol. 2022 Apr 28;13:802665. doi: 10.3389/fimmu.2022.802665 (PMC9095944; doi:10.3389/fimmu.2022.802665)

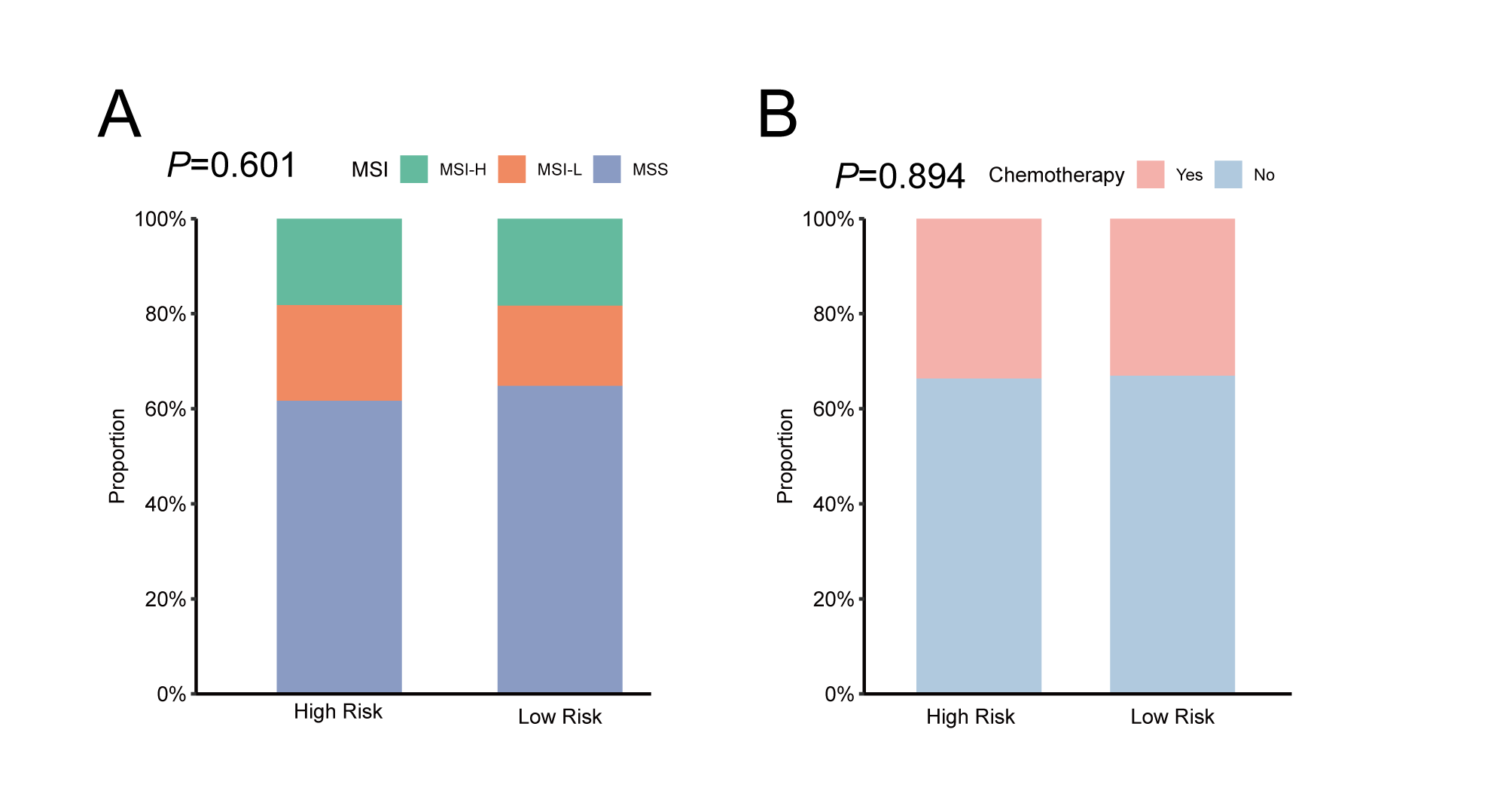

Supplement: Supplementary Figure 1 — Relationship between MSI status, chemotherapy in IRGPI groups.(A) MSI status. (B) Chemotherapy. MSI, microsatellite instability. [file Image_1.tif]
