# Supplementary material for: Identification of a Novel Immune Landscape Signature for Predicting Prognosis and Response of Colon Cancer to Immunotherapy
Source: Front Immunol. 2022 Apr 28;13:802665. doi: 10.3389/fimmu.2022.802665 (PMC9095944; doi:10.3389/fimmu.2022.802665)
Supplement: Supplementary file 2 [file Table_1.docx]

Table S1 Relationship between EGFR and KRAS status in IRGPI groups

|  | EGFR status | | KRAS status | |
| --- | --- | --- | --- | --- |
|  | Mut | Non-mut | Mut | Non-mut |
| IRGPI-high group | 13 | 213 | 80 | 146 |
| IRGPI-low group | 8 | 219 | 84 | 143 |
| *P-*value | 0.259 | | 0.722 | |
